# Supplementary figures and images for: Rational engineering of industrial S. cerevisiae: towards xylitol production from sugarcane straw
Source: J Genet Eng Biotechnol. 2022 May 25;20:80. doi: 10.1186/s43141-022-00359-8 (PMC9133290; doi:10.1186/s43141-022-00359-8)

**A**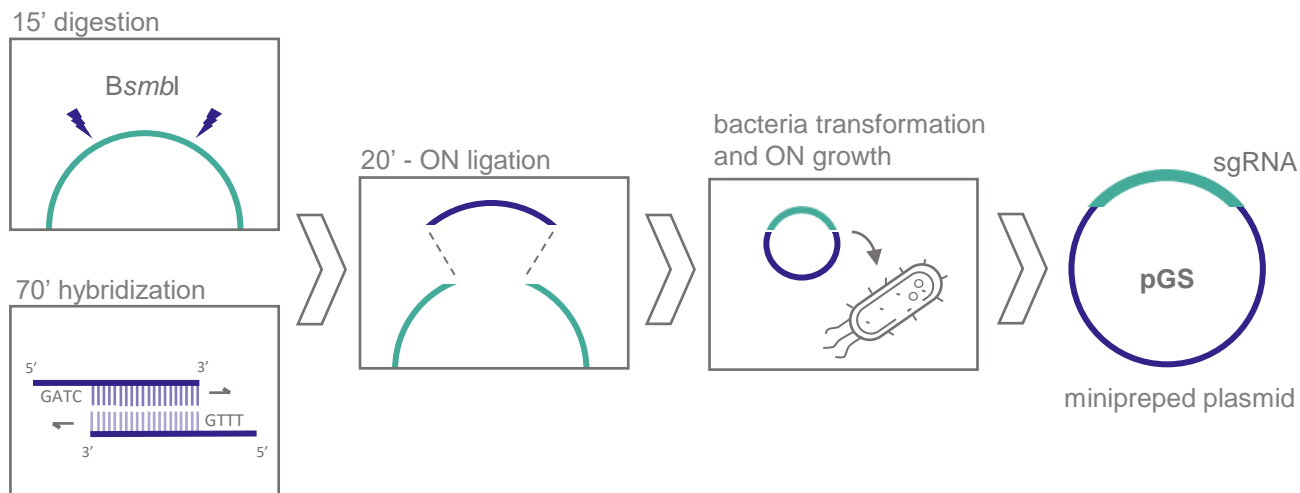**B**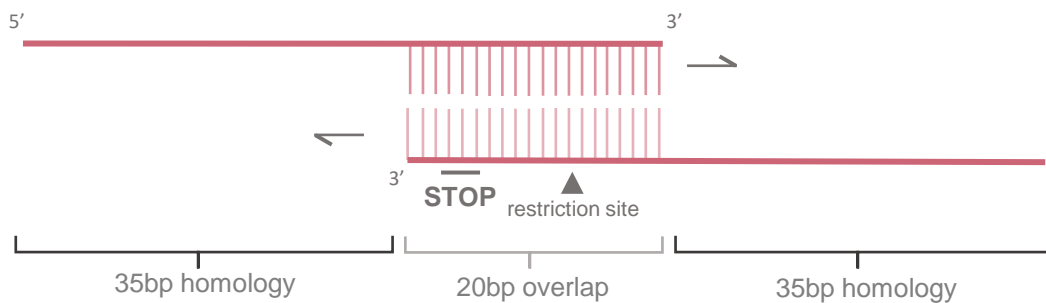**C**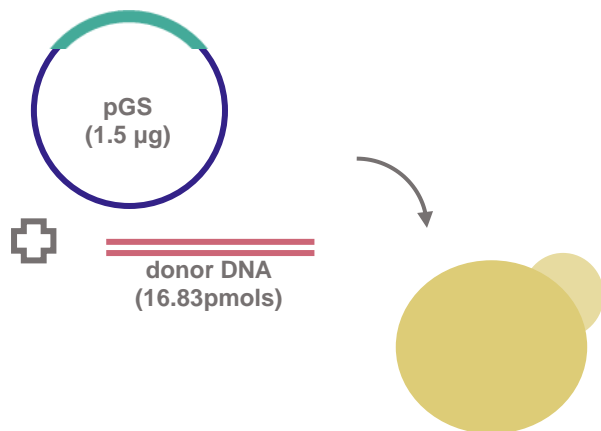

Supplement: Supplementary file 4 — Additional file 4: Supplementary Figure S1. Cloning procedures for using pGS in a CRISPR-Cas9 editing event. [file 43141_2022_359_MOESM4_ESM.pdf]

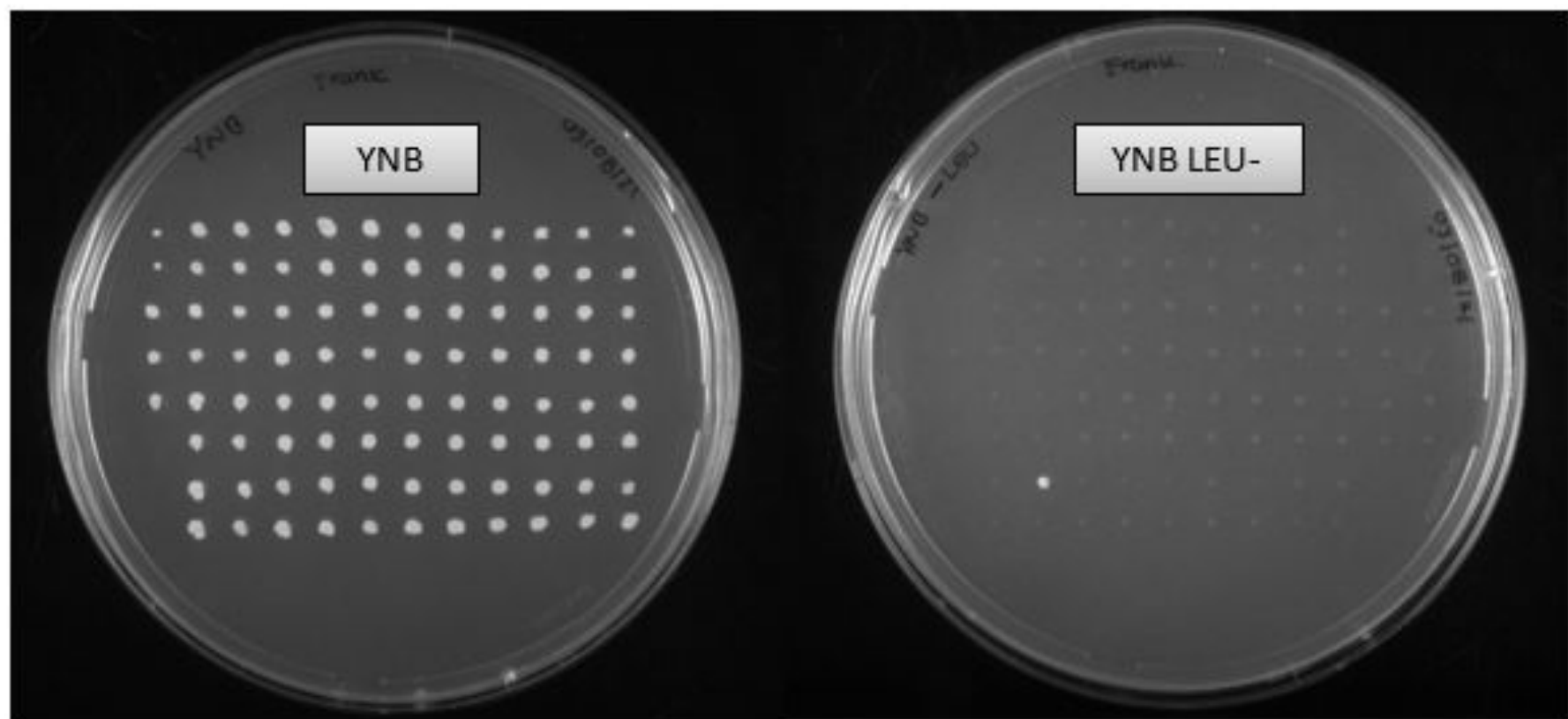

Supplement: Supplementary file 5 — Additional file 5: Supplementary Figure S2. Transformation efficiency of the LEU2 locus knockout in LVY34.4 (PE-2, MATa). [file 43141_2022_359_MOESM5_ESM.pdf]

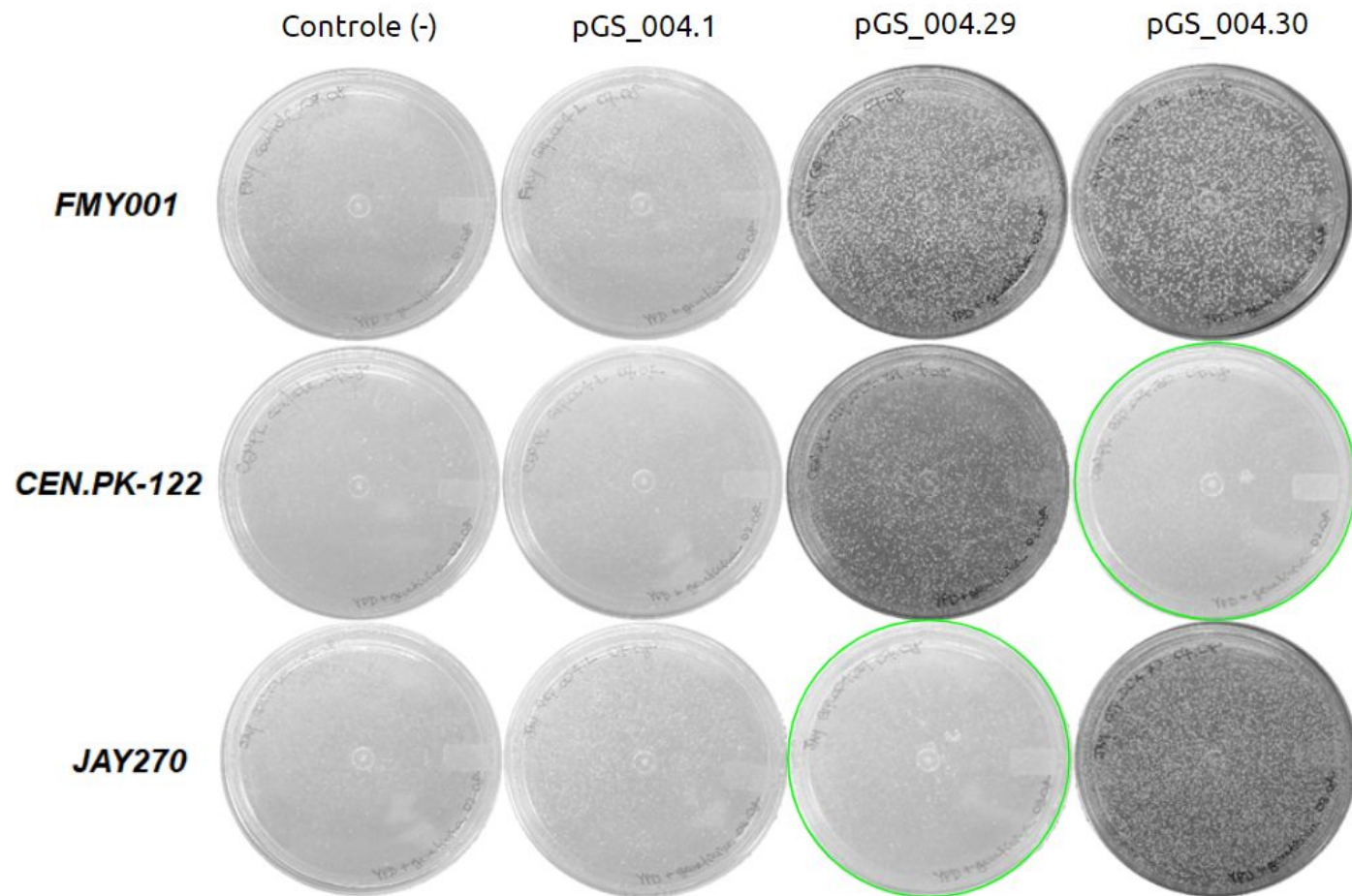

Supplement: Supplementary file 6 — Additional file 6: Supplementary Figure S3. Testing of sgRNAs targeting the HO locus in strains CEN.PK-122, SA-1 and PE-2. [file 43141_2022_359_MOESM6_ESM.pdf]

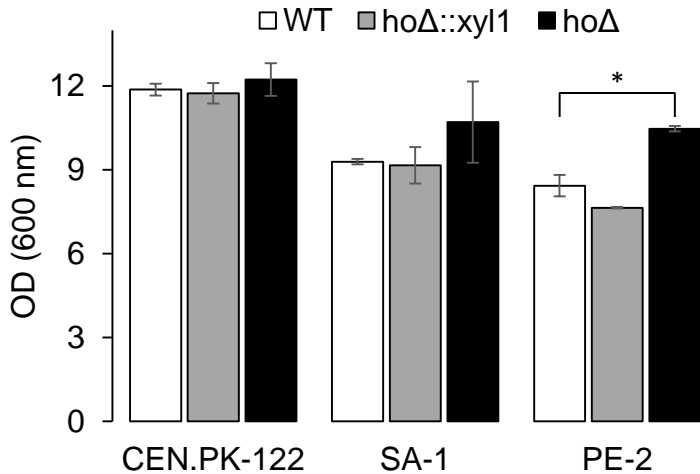

Supplement: Supplementary file 7 — Additional file 7: Supplementary Figure S4. Testing of the fitness of strains PE-2, SA-1 and CEN.PK-122 with a hoΔ genotype. [file 43141_2022_359_MOESM7_ESM.pdf]

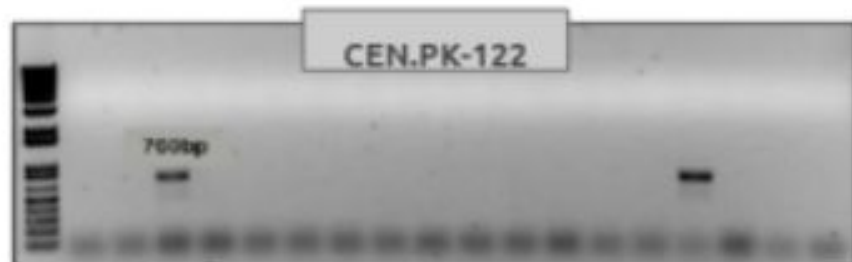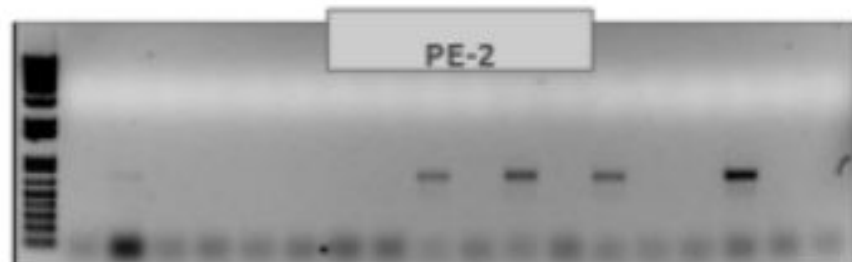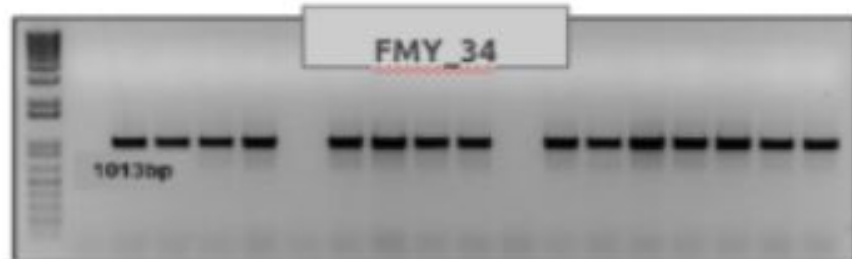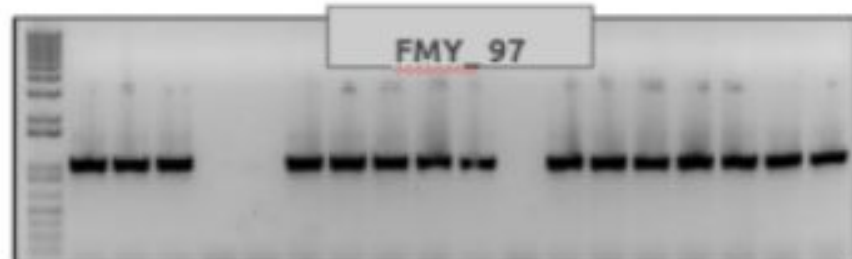

Supplement: Supplementary file 8 — Additional file 8: Supplementary Figure S5. Editing efficiency of strains PE-2, CEN.PK-122 and FMY097/FMY034 (SA-1 segregants) for integration of a xylose reductase cassette. [file 43141_2022_359_MOESM8_ESM.pdf]
